# Testicular cancer presenting as disseminated tuberculosis: A case report

**DOI:** 10.1016/j.amsu.2021.102975

**Published:** 2021-11-11

**Authors:** Faris Najdawi, Matthew Means, Ryan Didde, Michael Feloney

**Affiliations:** a Creighton University School of Medicine Department of Urology, 7710, Mercy Road, Suite 501, USA; b Des Moines University, 3200 Grand Ave, Des Moines, IA, 50312, USA

**Keywords:** NSGCT, Nonseminomatous germ cell tumor, NSGCT, Case report, Germ cell tumor, Tuberculosis, Tuberculous epididymo-orchitis

## Abstract

Introduction and Importance: Tuberculosis is one of the leading infectious causes of mortality worldwide. In the United States, foreign-born persons account for 70% of tuberculosis (TB) diagnoses. Comparatively, testicular cancer is much less common. However, metastatic disease may present similarly. Diagnosis is supported by elevated tumor markers and radical orchiectomy with specimen biopsy confirms the diagnosis and tumor type. Following resection, adjuvant treatment for metastatic disease includes chemotherapy.

Case Presentation: This case describes a 22-year-old male immigrant with shortness of breath as the presenting symptom. Chest imaging showed a cavitary lung lesion encroaching the bronchus and left atrium. The patient was placed on airborne precautions and a complex hospital course ensued which resulted in the diagnosis of metastatic nonseminomatous germ cell tumor. The patient's 8 cm testicular tumor was treated with radical orchiectomy followed by chemotherapy. His condition deteriorated quickly, and he passed away in the hospital.

Clinical Discussion: Metastatic testicular cancer is relatively rare compared to tuberculosis, especially in the immigrant population. Differentiating extrapulmonary TB from metastatic disease can pose a diagnostic challenge due to similar presentations. Complete physical exam including the genitalia is paramount in discerning a diagnosis of testicular cancer.

Conclusion: Incidence of metastatic testicular cancer is much less common than extrapulmonary tuberculosis but must always be included on the differential for a young male.

## Highlights

• Though relatively rare, testicular cancer is the most common malignancy among men 20–40 years old in the United States.
• In any young man with a persistent cough, metastatic testicular cancer must be evaluated and excluded.
• Complete physical exam, including genitalia, is vital following discovery of a lung lesion, especially in a young male.
• Delay in diagnosis of testicular cancer may delay treatment resulting in worse patient outcomes.

## 1 Introduction

Tuberculosis (TB) is a leading infectious cause of mortality worldwide with an estimated 10 million cases in 2020 [1]. In the United States, TB has an incidence of 26 per 100,000 persons with foreign-born persons accounting for 70% of TB diagnoses in 2019 [2]. The disease most commonly infects the lungs (pulmonary TB) but can also affect other organs (extrapulmonary TB). The combination of fevers, night sweats, and hemoptysis is a classic presentation of pulmonary TB, and a scrotal mass may be present in tuberculous epididymo-orchitis. In contrast, testicular cancers are relatively rare with an incidence of 1.7 per 100,000 persons and have a higher incidence in non-Hispanic whites compared to Hispanic persons [3,4]. In this study, we report a unique presentation of a metastatic mixed nonseminomatous germ cell tumor (NSGCT) mimicking disseminated tuberculosis in a young adult male early in the year 2021.

This work has been reported in line with the SCARE 2020 criteria [5].

## 2 Presentation of case

A 22-year-old Guatemalan male presented to the emergency department (ED) with left-sided chest pain and shortness of breath. He reported his symptoms began four months prior and progressively worsened. He also described intermittent hemoptysis, fevers, night sweats, and a 20 lbs weight loss over those four months. The patient had no past medical history, no history of surgeries, no known genetic abnormalities, and no family history of cancer. He had no history of drug use or allergies but had smoked cigarettes for two years before recently quitting. He had been working in construction since his arrival to the United States 18 months prior.

**Fig. 1:**
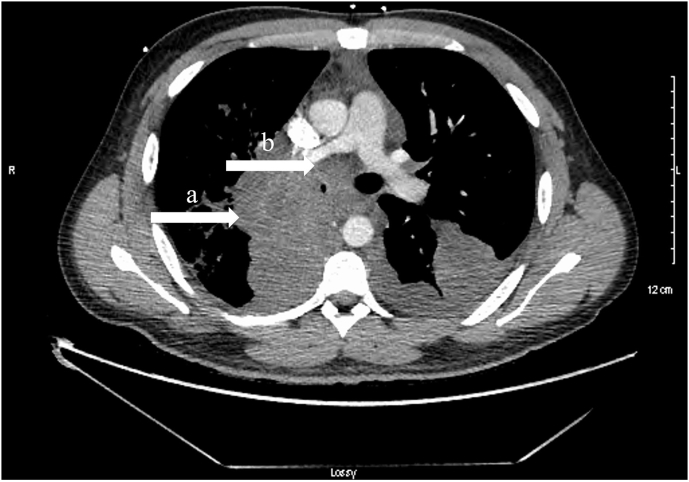
Very large area of opacification in the right perihilar region measuring 12 × 6 cm shape (a) and right lung base involving mediastinum and transecting right bronchus (b).

On the initial exam, the patient was tachycardic and tachypneic. A chest X-ray revealed a right upper lobe consolidation with moderate bilateral pleural fluid. Computed tomography angiogram (CTa), conducted using LightSpeed VCT 64-slice CT system, showed large mass-like consolidation in the right perihilar region measuring 12 × 6 cm and involving the mediastinum, an area of cavitation transecting the right mainstem bronchus, and mediastinal lymphadenopathy (see Fig. 1). An addendum to the radiology report later raised the concern of a large mass extending into the left atrium, warranting immediate thoracic surgery consult. Initial laboratory workup in the ED revealed a white blood cell count of 21.8 with 84% neutrophils, hemoglobin 9.7, platelets 489, lactic acid 1.7, C-reactive protein 304, and negative troponins. Urinalysis revealed urobilinogen without blood or pyuria. Laboratory tests for COVID-19, influenza, RSV, and HIV were all negative. At the time of initial ED presentation, the patient met institutional criteria for sepsis and was started on broad-spectrum antibiotics.

QuantiFERON-TB gold testing, acid-fast bacteria sputum cultures, and blood cultures were taken. Transthoracic echocardiogram (TTE) and bronchial biopsies were recommended before surgical intervention for the lung mass invading the left atrium. TTE revealed a 3.4 cm × 2.1 cm left atrial mass. Bronchoscopy showed an endobronchial mass occluding the middle and lower lobes and needle biopsies were taken. Reevaluation of the patient revealed an enlarged, nonpainful testicle. On further questioning, the patient noted the mass had been there for several months, at which point urology was consulted.

**Fig. 2:**
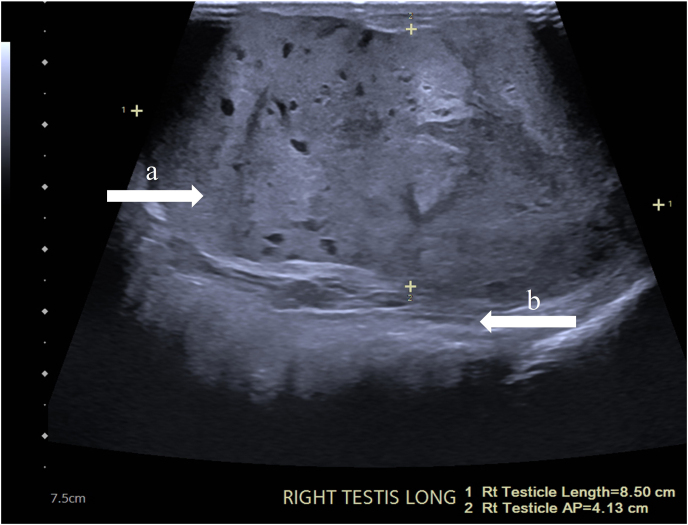
Long axis of right testicle showing complex solid mass measuring 8.5 × 4.1 × 4.7 cm (a), extending beyond tunica albuginea (b). Initial CTa on 1/5/21.

Scrotal ultrasound using grey scale and color doppler revealed an 8.5 cm × 4.1 cm x 4.7 cm complex right testicular mass and a normal left testicle (see Fig. 2). Alpha-fetoprotein serum concentration was found to be elevated at 3519.1 ng/mL, beta-human chorionic gonadotropin at 217 mIU/mL, and lactate dehydrogenase at 866 U/L. Due to imaging and laboratory concerns for testicular cancer, radical orchiectomy was performed on hospital day four. The procedure was performed by a professor of urology with over 20 years of surgical experience at an academic hospital. Histopathology of the endobronchial mass revealed germ cell tumor (GCT) consistent with metastasis of primary testicular yolk sac tumor, while the testicular specimen was found to be 80% yolk sac tumor and 20% embryonal carcinoma with lymphovascular invasion. Repeat CT abdomen/pelvis showed a known left atrial filling defect, no retroperitoneal lymphadenopathy, and a 2 cm right renal mass concerning for metastasis, confirming a diagnosis of stage IIIc NSGCT.

The patient was started on a 5-day course of bleomycin, etoposide, and cisplatin (BEP) without further surgical intervention at that time due to the large tumor burden and cardiac involvement.

The patient tolerated the treatment poorly, developing hypoxia, acute kidney failure, and neutropenia. Following completion of his 5-day BEP, a post-chemotherapy transesophageal echocardiogram and CT scan showed a significant decrease in tumor size. Despite this, the patient's condition deteriorated. Adjuvant external beam radiation was contraindicated at this time due to the patient's critical condition. He developed acute respiratory distress syndrome necessitating ECMO, then acute renal failure warranting continuous renal replacement therapy. The patient ultimately experienced acute hepatic failure and expired. An autopsy request was declined by the family. His body was transported to Guatemala for burial shortly after his passing.

## 3 Discussion

This case is unique as it presents a diagnostic dilemma for the treatment team regarding an infectious versus neoplastic etiology. Given the patient's recent immigration from a TB-endemic country, infectious etiologies must be ruled out in the differential diagnosis. The patient's productive cough with intermittent hemoptysis, imaging finding of cavitary upper lobe lung lesions, and lack of enlarged retroperitoneal lymph nodes all support tuberculosis as a potential diagnosis. Reasons against possible disseminated tuberculosis include an immunocompetent state (HIV-negative) and no recent sick contacts.

While TB has an incidence of 20.7 per 100,000 in Guatemala, testicular germ cell tumors are relatively rare, with rates per 100,000 of 0.6 in Guatemala and 3.9 among US Hispanics [6,7]. However, GCTs are the most diagnosed malignancy in US males between 15 and 44 years old [3]. Comprising 95% of testicular tumors, GCTs consist of multiple subtypes, the most common being seminoma. Mixed germ cell, the second most common subtype, was seen in this patient and may consist of various proportions of embryonal, choriocarcinoma, teratoma, and yolk sac types [8]. Staging is critical in evaluating the prognosis and treatment modalities of testicular cancer. Primary lymphatic sites of metastatic testicular disease are inter-aortocaval lymph nodes, and CT scans detect between 70 and 80% of positive nodes [[9], [10], [11]]. This case report is perplexing because it does not follow the usual pathway for metastatic disease. After radical orchiectomy, adjuvant treatment for metastatic NSGCTs consists of BEP chemotherapy which is highly effective in lower-stage malignancies. Patients with metastatic disease are classified according to the International Germ Cell Cancer Collaborative Group as Good, Intermediate, or Poor Risk with a 5-year survival of 92%, 72%, and 48%, respectively [12]. At the time of the patient's diagnosis, distant metastases were present, and his condition was classified as Poor Risk, a stratification present in only 16% of NSGCTs [13].

Though testicular cancer metastasis to the mediastinum is well documented, it is imperative to keep in mind extragonadal GCT without primary testicular involvement occurs in 2–5% of cases [14,15]. These neoplasms typically have worse prognoses with a 5-year survival rate of 45% [15].

The SCARE 2020 paper was used in the development of this paper [4].

## 4 Conclusion

Symptoms of TB can cause other disease processes, such as metastatic testicular cancer, to be overlooked, posing a diagnostic challenge. Diagnosis of testicular cancer is based on a thorough history and pathologic examination. Incidence of metastatic disease is much less common than tuberculosis but must always be included in the differential for a young male. An initial more thorough examination, especially of genitalia, could have expedited the correct diagnosis and initiation of treatment, possibly saving his life.

## Patient perspective

Continuing to educate young men on testicular self-examination through community outreach programs may help raise awareness for this disease.

## Provenance and peer review

Not commissioned, externally peer-reviewed.

## Declaration of competing interest

Written informed consent was obtained from the patient for publication of this case report and accompanying images. A copy of the written consent is available for review by the Editor-in-Chief of this journal on request.

## Registration of research studies

Name of the registry: None.

Unique Identifying number or registration ID: None.

Hyperlink to your specific registration (must be publicly accessible and will be checked): None.
