# Supplementary material for: Testicular cancer presenting as disseminated tuberculosis: A case report
Source: Ann Med Surg (Lond). 2021 Nov 11;72:102975. doi: 10.1016/j.amsu.2021.102975 (PMC8599095; doi:10.1016/j.amsu.2021.102975)
Supplement: Multimedia component 1 [file mmc1.docx]

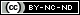
** CARE Checklist (2013) of information to include when writing a case report**

| **Topic** | **Item** | **Checklist item description** | **Reported on Page** |
| --- | --- | --- | --- |
| **Title** | **1** | The words “case report” should be in the title along with the area of focus . . . . . . . . . . . . . . . . . . . . . . . . . . . . . . . . . . . | **X** |
| **Key Words** | **2** | 2 to 5 key words that identify areas covered in this case report. . . . . . . . . . . . . . . . . . . . . . . . . . . . . . . . . . . . . . . . . . . . . . . . . . . . . . . | **X** |
| **Abstract** | **3a** | Introduction—What is unique about this case? What does it add to the medical literature? . . . . . . . . . . . . . . . . . . . . | **X** |
|  | **3b** | The main symptoms of the patient and the important clinical findings . . . . . . . . . . . . . . . . . . . . . . . . . . . . . . . . . . . . . . | **X** |
|  | **3c** | The main diagnoses, therapeutics interventions, and outcomes . . . . . . . . . . . . . . . . . . . . . . . . . . . . . . . . . . . . . . . . . . . | **X** |
|  | **3d** | Conclusion—What are the main “take-away” lessons from this case? . . . . . . . . . . . . . . . . . . . . . . . . . . . . . . . . . . . . . | **X** |
| **Introduction** | **4** | One or two paragraphs summarizing why this case is unique with references . . . . . . . . . . . . . . . . . . . . . . | **X** |
| **Patient Information** | **5a** | De-identified demographic information and other patient specific information . . . . . . . . . . . . . . . . . . . . . . . . | **X** |
|  | **5b** | Main concerns and symptoms of the patient . . . . . . . . . . . . . . . . . . . . . . . . . . . . . . . . . . . . . . . . . . . . . . . . . . . . . . . | **X** |
|  | **5c** | Medical, family, and psychosocial history including relevant genetic information (also see timeline). . . | **X** |
|  | **5d** | Relevant past interventions and their outcomes . . . . . . . . . . . . . . . . . . . . . . . . . . . . . . . . . . . . . . . . . . . . . . . . . . . | **X** |
| **Clinical Findings** | **6** | Describe the relevant physical examination (PE) and other significant clinical findings. . . . . . . . . . . . . . . . | **X** |
| **Timeline** | **7** | Important information from the patient’s history organized as a timeline . . . . . . . . . . . . . . . . . . . . . . . . . | **X** |
| **Diagnostic Assessment** | **8a** | Diagnostic methods (such as PE, laboratory testing, imaging, surveys). . . . . . . . . . . . . . . . . . . . . . . . . . . . . . | **X** |
|  | **8b** | Diagnostic challenges (such as access, financial, or cultural) . . . . . . . . . . . . . . . . . . . . . . . . . . . . . . . . . . . . . . . | **X** |
|  | **8c** | Diagnostic reasoning including other diagnoses considered . . . . . . . . . . . . . . . . . . . . . . . . . . . . . . . . . . . . . . . . | **X** |
|  | **8d** | Prognostic characteristics (such as staging in oncology) where applicable . . . . . . . . . . . . . . . . . . . . . . . . . . | **X** |
| **Therapeutic Intervention** | **9a** | Types of intervention (such as pharmacologic, surgical, preventive, self-care) . . . . . . . . . . . . . . . . . . . . . . . . . . . . . . . | **X** |
|  | **9b** | Administration of intervention (such as dosage, strength, duration) . . . . . . . . . . . . . . . . . . . . . . . . . . . . . . . . . . . . . . . . | **X** |
|  | **9c** | Changes in intervention (with rationale) . . . . . . . . . . . . . . . . . . . . . . . . . . . . . . . . . . . . . . . . . . . . . . . . . . . . . . . . . . . . . . . | **X** |
| **Follow-up and**  **Outcomes** | **10a** | Clinician and patient-assessed outcomes (when appropriate) . . . . . . . . . . . . . . . . .. . . . . . . . . . . . . . . . . . . . . . . . . . . . | **X** |
|  | **10b** | Important follow-up diagnostic and other test results . . . . . . . . . . . . . . . . . . . . . . . . . . . . . . . . . . . . . . . . . . . . . . . . . . . . | **X** |
|  | **10c** | Intervention adherence and tolerability (How was this assessed?) . . . . . . . . . . . . . . . . . . . . . . . . . . . . . . . . . . . . . . . . . | **X** |
|  | **10d** | Adverse and unanticipated events . . . . . . . . . . . . . . . . . . . . . . . . . . . . . . . . . . . . . . . . . . . . . . . . . . . . . . . . . . . . . . . . . . . | **X** |
| **Discussion** | **11a** | Discussion of the strengths and limitations in your approach to this case . . . . . . . . . . . . . . . . . . . . . . . . . . . . . . . . . . . | **X** |
|  | **11b** | Discussion of the relevant medical literature. . . . . . . . . . . . . . . . . . . . . . . . . . . . . . . . . . . . . . . . . . . . . . . . . . . . . . . . . | **X** |
|  | **11c** | The rationale for conclusions (including assessment of possible causes) . . . . . . . . . . . . . . . . . . . . . . . . . . . . . . . . . . . | **X** |
|  | **11d** | The primary “take-away” lessons of this case report . . . . . . . . . . . . . . . . . . . . . . . . . . .. . . . . . . . . . . . . . . . . . . . . . . . | **X** |
| **Patient Perspective** | **12** | When appropriate the patient should share their perspective on the treatments they received . . . . . . . . . . . . . . . . . | **X** |
| **Informed Consent** | **13** | Did the patient give informed consent? Please provide if requested . . . . . . . . . . . . . . . . . . . . . . . . . . . . . . . . . . . . . . | **Yes**  **No** |
